# Abulia following penetrating brain injury during endoscopic sinus surgery with disruption of the anterior cingulate circuit: Case report

**DOI:** 10.1186/1471-2377-6-4

**Published:** 2006-01-23

**Authors:** Alexander A Grunsfeld, Ivan S Login

**Affiliations:** 1 Department of Neurology, University of Virginia Health Sciences Center, Charlottesville, Virginia, 22908, USA

## Abstract

**Background:**

It is common knowledge that the frontal lobes mediate complex human behavior and that damage to these regions can cause executive dysfunction, apathy, disinhibition and personality changes. However, it is less well known that subcortical structures such as the caudate and thalamus are part of functionally segregated fronto-subcortical circuits, that can also alter behavior after injury.

**Case presentation:**

We present a 57 year old woman who suffered penetrating brain injury during endoscopic sinus surgery causing right basal ganglia injury which resulted in an abulic syndrome.

**Conclusion:**

Abulia does not result solely from cortical injury but can occur after disruption anywhere in the anterior cingulate circuit – in the case of our patient, most prominently at the right caudate.

## Background

We present a patient who developed abulia following unilateral penetrating brain injury incurred during endoscopic sinus surgery (ESS). Major complications resulting from ESS are uncommon and include intraorbital soft tissue and ocular damage and intracranial infection, hemorrhage and CSF leak. Of 2108 ESS patients only 0.47% had intracranial injury with 0.85% incidence of all major complications [1]. This case illustrates that injury to subcortical structures may disrupt behaviors commonly assigned to control by the frontal lobes.

## Case presentation

This otherwise healthy 57 year old woman was referred to an otolaryngologist for evaluation of chronic ear infections and recurrent sinusitis. The otolaryngologist diagnosed chronic sinusitis based on the clinical exam and CT imaging. The planned surgery included bilateral endoscopic ethmoidectomy, bilateral middle meatus antrostomy and left inferior turbinate cautery.

During the right posterior ethmoid exploration, a 2 mm tissue fragment was removed for histopathological analysis. Cerebrospinal fluid was observed leaking from the surgical biopsy site. The apparent opening in the cribriform plate was sealed with gelfoam and the remainder of the procedure completed.

**Figure 1:**
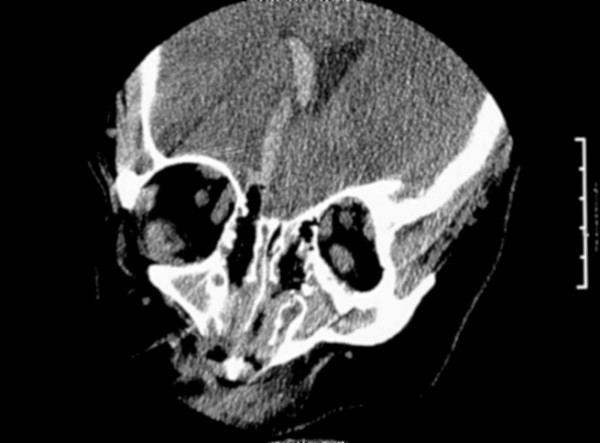
Coronal CT; blood and air extending in a straight line from the cribriform plate to the right lateral ventricle, with blood filling the right lateral ventricle. (Note: blood in the third ventricle was also seen but not shown here.)

With concern for a CSF leak an unenhanced CT was obtained post-operatively revealing intracranial penetration and hemorrhage (Figure 1). The 2 mm tissue sample obtained during the procedure was identified as normal cerebral white matter. In the recovery room, the patient awakened without difficulty in "satisfactory condition with intact vision."

Neurosurgical consultation one day after surgery described slight drowsiness and minimal left upper extremity drift. She was empirically started on fosphenytoin and antibiotics. The following day her drift had resolved and she appeared less lethargic.

The remainder of the hospitalization was uncomplicated and she was discharged seven days later after completing her course of antibiotics. She took valproic acid for seizure prophylaxis for several days at home, but this was discontinued due to adverse effects.

**Figure 2:**
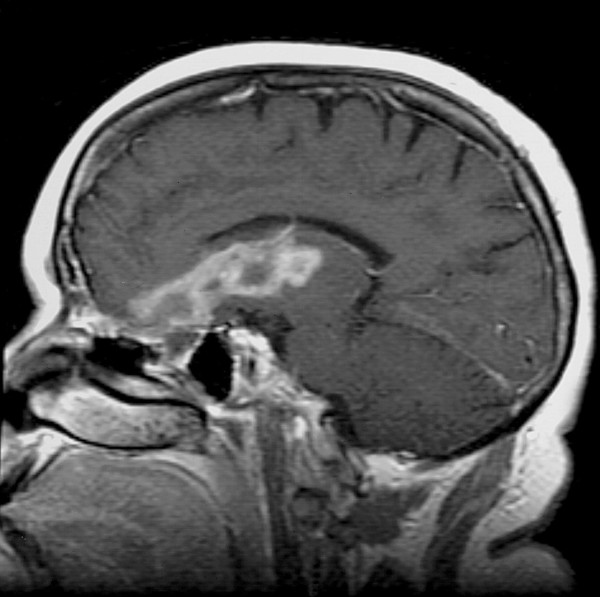
Sagittal T1 Gadolinium-enhanced MRI; evolution of blood products in the right inferior frontal lobe extending to the basal ganglia.

**Figure 3:**
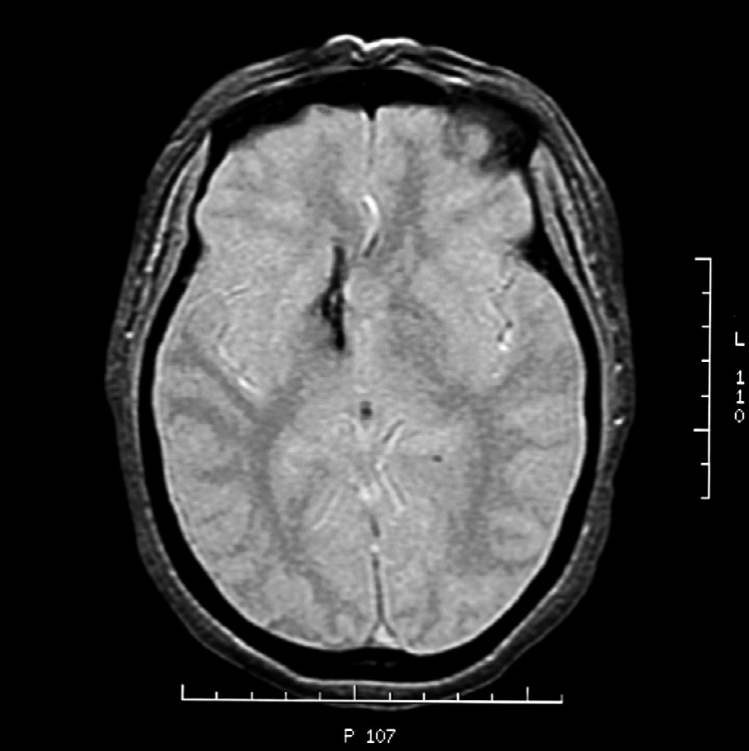
Axial Gradient Echo MRI; this image demonstrates a line of injury through the basal ganglia.

An MRI scan two weeks later (Figure 2) documented evolution of the hemorrhage. Restricted diffusion along the course of the injury (image not shown) indicated tissue infarction. A brain MRI three months after surgery (Figure 3) revealed resolution of the hemorrhage with residual encephalomalacia along the path of injury involving the right caudate, medial internal capsule and anterior right thalamus.

Over the ensuing months the family reported that the patient's personality had changed. They described her as apathetic, mildly depressed, emotionally labile and irritable. She herself complained of having no energy and seemed to have lost interest in life and those activities that had previously stimulated her. Prior to the surgery she had worked full time as an aide in a center for learning disabled children plus an additional 20 hours weekly as an aide in a home health agency. She also was involved extensively with her church and community activities. After recovering from the surgery she was unable to return to work. She would sit at home and do nothing. She did not even wish to drive her car any longer. She also felt that her cognitive abilities had declined significantly. She complained of difficulty with attention and concentration. Although she reported no decline in memory, her family did notice that she was more forgetful.

The patient was evaluated 6 months after the injury by a rehabilitation neuropsychiatrist who found that she was apathetic, had "blunted and constricted affect" and specifically showed "no evidence of neuro-vegetative symptoms of depression." Based on this assessment, the neuropsychiatrist actually discontinued Prozac treatment started by other physicians and initiated therapy instead with amantadine. The same clinician continued to interact with her until 28 months after surgery and never observed any clinically significant depression. More extensive neuropsychological testing was performed at a different facility 32 months after surgery and concluded again, that the patient's primary cause of cognitive impairment and resultant loss of functionality resulted from an abulic syndrome and not depression. While the patient scored for mild to moderate depression on the Beck Depression Inventory-11, most of the score was based on symptoms that coexist with abulia such as decreased interest in people or activities, loss of pleasure, decreased energy, concentration, motivation and initiative. However, the patient actually scored low on symptoms that are exclusive to depression such as feelings of sadness, worthlessness, guilt and hopelessness. Her apathy, lack of motivation and initiative were the changes that dramatically stood out. Thus it was possible to distinguish clearly between abulia and depression. The ultimate conclusion was that our patient had primary abulia with some depression secondary to her self-perceived change in personality. This fit well with her own assessment. She described her mood as a response to the different person she had become and her inability to live life as she was once able. These neuropsychological tests also revealed other complex functions affected by the injury, namely "executive dysfunction and mild disinhibition." The abulia responded partially to amantadine, 100 mg daily, however, the patient reported that if she missed a few doses her difficulties with initiation and motivation would soon return.

## Conclusion

This case report highlights an interesting presentation of abulia following disruption of unilateral basal ganglia pathways. Abulia is characterized by apathy and lack of both motivation and goal-directed behavior. While widely known that frontal lobes modulate complex human behavior, basal ganglia involvement in such behaviors is less appreciated. However, in patients who were studied following basal ganglia lesions, abulia was seen, occurring most frequently in those with caudate lesions (unilateral and bilateral), but also following injury to the thalamus, lenticular nucleus and limbic circuits traversing the inferior internal capsule in the region of the genu [2-6]. These findings reflect the intimate anatomical and functional relationship between the basal ganglia and the frontal cortex.

Alexander et al [7] first described parallel organization of functionally segregated circuits linking basal ganglia and frontal cortex. Subsequent studies revealed the role of these circuits in human behavior – the dorsolateral prefrontal cortex in executive function, the orbitofrontal cortex in personality, social conduct and inhibition, and the anterior cingulate gyrus in motivational states [8]. Several other open circuits have since been identified within the fronto-subcortical pathways, as well as complex thalamocortical pathways completing and modulating the cortical-subcortical loops [9].

Our patient illustrates the consequence of disruption of fronto-subcortical circuits at the level of the basal ganglia, although some degree of cortical involvement cannot be ruled out given the path of injury. The anterior cingulate circuit appears especially relevant in this case given the predominance of abulic symptoms. It is comprised of the anterior cingulate cortex (Brodmann's area 24) – the region enveloping the rostrum of the corpus callosum – and its projections to the ventral striatum which includes the ventromedial caudate. The loop continues with ventral striatum connections to the ventral pallidum which connects to the ventral anterior nucleus of the thalamus. The circuit is closed with projections back to the anterior cingulate cortex and reciprocal projections to other cortical areas [10].

This circuit is essential for the initiation of behavior, motivation and goal orientation. Unilateral injury leads to abulia regardless of the side of injury [3,4] whereas bilateral anterior cingulate dysfunction leads to the severest form of abulia; akinetic mutism – a wakeful state characterized by marked apathy, mutism and lack of motor initiation [11]. In this state, there is minimal if any communication or response to the environment, including an apparent indifference to pain or discomfort. However, injury at any point in this circuit may produce an abulic syndrome. We propose that our patient suffered from abulia as a result of disruption of the anterior cingulate circuit, most prominently at the right caudate.

## Competing interests

The author(s) declare that they have no competing interests.

## Authors' contributions

AAG and ISL contributed equally to this work. Both authors read and approved the final manuscript.

## Pre-publication history

The pre-publication history for this paper can be accessed here:



## References

[B1] May M, Levine HL, Mester SJ, Schaitkin B (1994). Complications of endoscopic sinus surgery: Analysis of 2108 patients – Incidence and prevention. Layngoscope.

[B2] Bhatia KP, Marsden CD (1994). The behavioral and motor consequences of focal lesions of the basal ganglia in man. Brain.

[B3] Caplan LR, Schmahmann JD, Kase CS, Feldmann E, Baquis G, Greenberg JP, Gorelick PB, Helgason C, Hier DB (1990). Caudate infarcts. Arch Neurol.

[B4] Kumral E, Evyapan D, Balkir K (1999). Acute caudate vascular lesions. Stroke.

[B5] Etsuro M (2002). Impact of subcortical ischemic lesions on behavior and cognition. Annals NYAS Online.

[B6] Giroud M, Lemesle M, Madinier G, Billiar T, Dumas R (1997). Unilateral lenticular infarcts: radiological and clinical syndromes, aetiology and prognosis. JNNP.

[B7] Alexander MR, DeLong PL, Strick (1986). Parallel organization of functionally segregated circuits linking basal ganglia and cortex. Ann Rev Neurosci.

[B8] Cummings JL (1993). Frontal-subcortical circuits and human behavior. Arch Neurol.

[B9] McFarland NR, Haber SN (2002). Thalamic relay nuclei of the basal ganglia form both reciprocal and nonreciprocal cortical connections, linking multiple frontal cortical areas. J Neurosci.

[B10] Tekin S, Cummings JL (2002). Frontal-subcortical neuronal circuits and clinical neuropsychiatry. Journal of Psychosomatic Research.

[B11] Nemeth G, Hegedus K, Molnar L (1988). Akinetic mutism associated with bicingular lesions: clinicopathological and functional anatomical correlates. European Archives of Psychiatry and Neurological Sciences.

